# Biomolecular Chemistry in Liquid Phase Separated Compartments

**DOI:** 10.3389/fmolb.2019.00021

**Published:** 2019-04-03

**Authors:** Karina K. Nakashima, Mahesh A. Vibhute, Evan Spruijt

**Affiliations:** Institute for Molecules and Materials, Radboud University, Nijmegen, Netherlands

**Keywords:** coacervates, liquid-liquid phase separation, membraneless organelles, cytomimetic media, artificial cells

## Abstract

Biochemical processes inside the cell take place in a complex environment that is highly crowded, heterogeneous, and replete with interfaces. The recently recognized importance of biomolecular condensates in cellular organization has added new elements of complexity to our understanding of chemistry in the cell. Many of these condensates are formed by liquid-liquid phase separation (LLPS) and behave like liquid droplets. Such droplet organelles can be reproduced and studied *in vitro* by using coacervates and have some remarkable features, including regulated assembly, differential partitioning of macromolecules, permeability to small molecules, and a uniquely crowded environment. Here, we review the main principles of biochemical organization in model membraneless compartments. We focus on some promising *in vitro* coacervate model systems that aptly mimic part of the compartmentalized cellular environment. We address the physicochemical characteristics of these liquid phase separated compartments, and their impact on biomolecular chemistry and assembly. These model systems enable a systematic investigation of the role of spatiotemporal organization of biomolecules in controlling biochemical processes in the cell, and they provide crucial insights for the development of functional artificial organelles and cells.

## Cellular Organization by Liquid Phase Separated Compartments

Organization is a central theme in life across scales: from herds to individual organisms to cells (Saha and Galic, 2018). Subcellular organization plays an important role in both eukaryotic and prokaryotic cells: most cellular processes cannot be fully understood without taking into account the spatial distribution of molecules. In eukaryotes, organelles encased by a lipid membrane are key organizing elements, and they occupy a large fraction of the cellular volume (Heald and Cohen-Fix, 2014). In addition, many organelles that lack a membrane have been identified both in the nucleus and the cytoplasm of eukaryotic cells, suggesting that they offer additional advantages as a compartmentalization strategy (Mitrea and Kriwacki, 2016; Banani et al., 2017; Shin and Brangwynne, 2017). Examples include nucleoli, Cajal bodies and paraspeckles in the nucleus, and processing bodies and stress granules in the cytoplasm. Interestingly, not all membrane-free organelles are constitutively present, but assemble in response to the cell cycle state or to oxidative stress (Anderson and Kedersha, 2006; Smith et al., 2016; Alberti, 2017). However, much of their biological function, the factors that govern their assembly and their effect on biomolecular chemistry remain poorly understood. Here, we examine how *in vitro* models of membrane-free organelles can be used to address this blind spot in our knowledge of cellular organization.

The term membrane-free or membraneless organelles (MLOs) refers to a wide variety of subcellular bodies that lack a lipid boundary, with sizes in the order of 0.01–10 μm (Mitrea and Kriwacki, 2016; Aguilera-Gomez and Rabouille, 2017; Banani et al., 2017; Gomes and Shorter, 2018). Many of those bodies share other distinctive features: they are spherical, deform in flow and show wetting, dripping, and fusion. These are all characteristics of liquids, and increasing evidence suggests that many MLOs are, in essence, liquid droplets dispersed in the cytoplasm or nucleoplasm and formed through liquid-liquid phase separation (LLPS), although some are also reported to be gel-like solids (Brangwynne et al., 2015). In general, each MLO is enriched in a particular set of proteins, many of which contain intrinsically disordered regions (IDRs). Nucleic acids also frequently take part in MLO assembly (Zhang et al., 2017), or are taken up in already formed MLOs (Nott et al., 2016). Multiple weak interactions between blocks of charged or aromatic residues or between specific binding domains drive the condensation (Banani et al., 2017; Gomes and Shorter, 2018), while the lack of extensive secondary structures in the MLO-forming biomolecules is believed to be crucial in keeping the complexes dynamic, and thus liquid-like (Darling et al., 2018).

## Coacervates as Model Membraneless Organelles

In order to understand the functions of MLOs, systematic studies of their assembly, their physicochemical properties and their effect on biochemical processes are required. In cells, such studies are hampered, as, under stress, multiple biochemical pathways are activated, making it hard to determine for example whether MLO formation is a cause or consequence of the stress response (Alberti, 2017). *In vitro* models of MLOs offer an ideal platform to address these challenges. Such models must be designed to mimic two common aspects of most MLOs in cells: the liquid nature and the overall chemical and macromolecular composition. Both aspects can be realized in coacervates, which have long received attention as potential protocells that simulate the intracellular environment.

Coacervates are dense liquid droplets composed of macromolecules that separate from the dilute phase through LLPS either by segregation or association (van der Gucht et al., 2011; Aumiller and Keating, 2017). Simple coacervates are formed by maximizing favorable interactions between identical macromolecules (often polymers or proteins), thereby minimizing polymer-solvent interactions (segregation); complex coacervates are formed by maximizing favorable interactions between different types of macromolecules (association), such as polyelectrolytes of opposite charge. In either case, de-mixing produces droplets enriched in macromolecules that resemble the compartmentalized and crowded environment of MLOs. A wide range of macromolecules has been used to make coacervates, including combinations of synthetic polyelectrolytes (Spruijt et al., 2010; van der Gucht et al., 2011), polysaccharides (de Kruif et al., 2004) and peptides (Perry et al., 2015), or individual single-stranded nucleic acids (Jain and Vale, 2017; Merindol et al., 2018) and partially disordered proteins that are purified from MLOs in cells (Elbaum-Garfinkle et al., 2015; Feric et al., 2016; Nott et al., 2016). In many cases, the coacervates formed from proteins and/or RNA are so similar to MLOs that the latter could be termed “coacervate organelles”.

In this review, we argue that a fundamental understanding of MLOs and cellular organization calls for systematic studies of coacervate-based cytomimetic model systems. In particular, such studies should be used to shed light on three aspects of MLOs that are still poorly understood: (1) how can the assembly and dissolution of MLOs be controlled, (2) what rules govern the partitioning of biomolecules into MLOs, and (3) how are rates of reactions and other biochemical processes affected by MLOs? Here, we focus on progress made *in vitro* to answer these questions using cytomimetic model-MLOs, and we discuss opportunities for future steps. These developments will not only lead to a better understanding of living cells, but also help further advance the bottom-up assembly of synthetic cells, by providing them with internal organization and expanding their chemistry.

## Biochemical Control of Droplet Condensation and Dissolution

A cursory glance yields numerous similarities between MLO formation in cells and coacervate formation *in vitro*. Cells differ, however, in using active processes to achieve dynamic control over MLO assembly and disassembly (Falahati and Wieschaus, 2017). To understand these control mechanisms, we first consider the framework of liquid phase separation that underlies formation of coacervates and many MLOs. The condensation of *chain-like* macromolecules, such as IDPs, into a dense liquid phase *in vitro* is usually described by a mean-field Flory-Huggins theory (Brangwynne et al., 2015):

(1) $$\begin{array}{r} \frac{F}{k_{\text{B}}T}=\frac{\phi }{N}\ln\phi +\left(1-\phi \right)\ln\left(1-\phi \right)+F_{int} \end{array}$$

where *F* is the free energy, ϕ is the volume fraction, *N* is the chain length, and *F*_int_ is the interaction free energy. For simple coacervation, *F*_int_ is expressed using an effective macromolecule-solvent interaction parameter χ: *F*_int_ = χφ(1 − ϕ). For complex coacervation of polymers with an identical length and charge density (σ), *F*_int_ can be expressed by a Debye-Hückel approximation using an electrostatic interaction constant α: $F_{\mathrm{int}}=\alpha {\left(\text{σφ}\right)}^{3/2}$.

**Figure 1:**
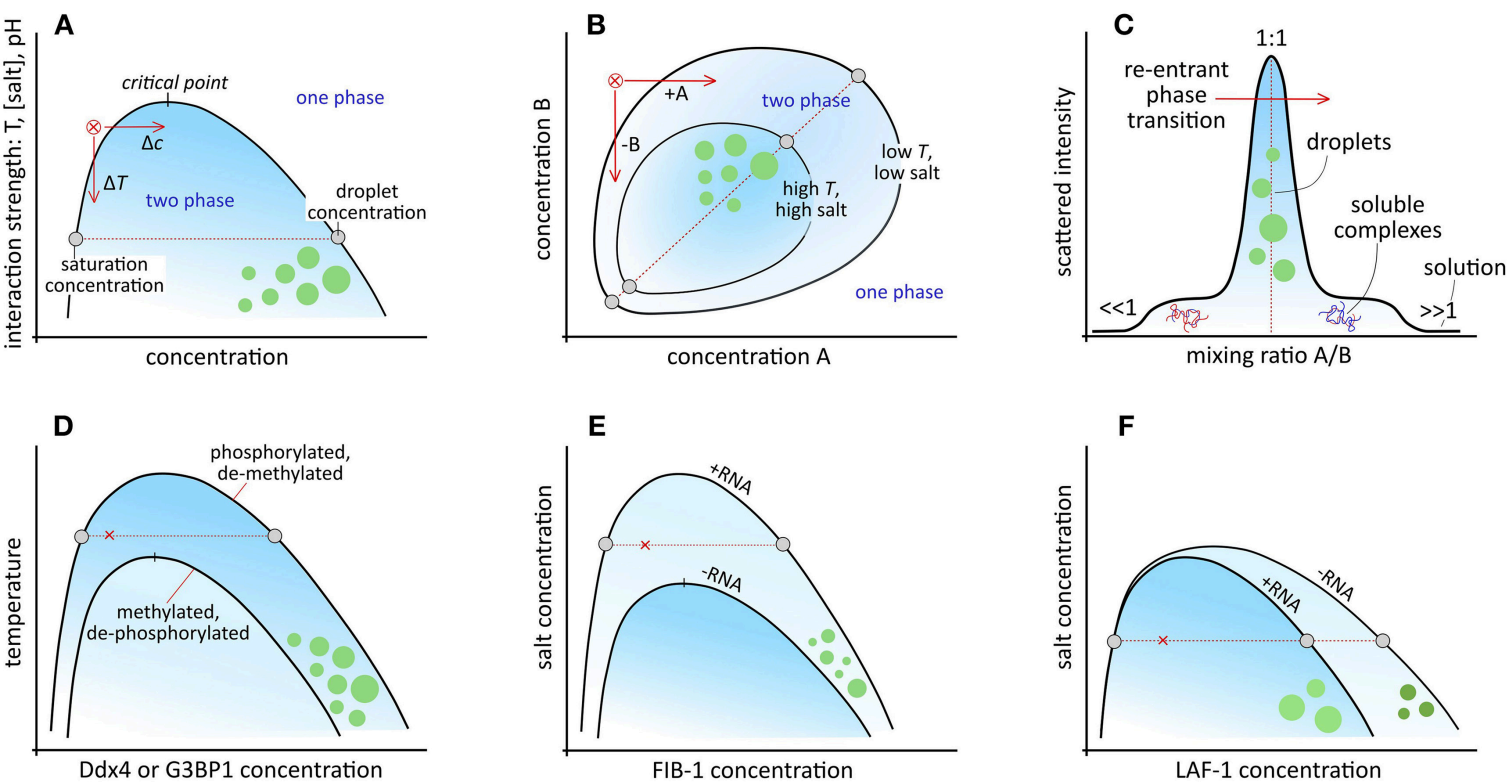
Schematic phase diagrams for: **(A)** simple and symmetric complex coacervates with coexistence between a saturated dilute phase and a concentrated droplet phase. Condensation from the red cross can be induced by increasing the concentration (Δ*c*) or lowering the temperature (Δ*T*), or salt concentration. Frequently, only the low-concentration branch of the binodal, or coexistence curve is shown. **(B)** Non-symmetric complex coacervates. Condensation can be induced by increasing the concentration of A, or reducing the concentration of B. **(C)** Cross-section through the two-phase region in **(B)** for varying mixing ratio, highlighting the re-entrant phase transition from one phase, via soluble complexes to two phases and back to one phase. **(D)** Control over coacervation by reversible post-translational modifications, like in Ddx4 and G3BP1. **(E)** RNA-dependent coacervation in FIB-1. **(F)** Effect of RNA on the density and viscosity of the coacervate phase of LAF-1.

When the interactions are sufficiently strong (large, negative χ, large α, or large σ), a first-order phase transition is predicted, resulting in two coexisting liquid phases: a dense (coacervate) phase and a dilute phase (Figure 1A). The width of the two-phase region is set by the relative interaction strength (χ or ασ^3/2^), which is in general a function of temperature, pH, salt concentration, and the chemical groups in the macromolecules (van der Gucht et al., 2011; Brangwynne et al., 2015). *In vitro* model MLOs are generally responsive to changes in one or more of these parameters: they have been assembled and dissolved by temperature (Nott et al., 2015; Quiroz and Chilkoti, 2015), pH (Kaibara et al., 2000; Koga et al., 2011), cosolvents (Simon et al., 2017), and salt (Spruijt et al., 2010; Nakashima et al., 2018).

The mean-field approach described above is highly simplistic, and does not take into account many factors that can affect coacervation, such as sequence specificity (Pak et al., 2016; Dignon et al., 2018; Langdon et al., 2018), charge correlation (Nott et al., 2015; Chang et al., 2017), and soluble complex formation (Delaney and Fredrickson, 2017). More complex theories of coacervation that account for many of these factors, as shown by numerical simulations, have recently been developed (Delaney and Fredrickson, 2017; Sing, 2017; Zhou et al., 2018). However, none of these provides a quantitative explanation for all types of liquid phase separating proteins and polymers, and the relative simplicity of the classical mean-field model, which can provide semi-quantitative agreement with experimental phase diagrams based on a single effective interaction parameter (χ or α), is therefore still attractive (Brangwynne et al., 2015; Nott et al., 2015; Wei et al., 2017).

One common feature between most LLPS models is that they describe the macromolecules as polymeric chains. Chain flexibility is known to have a very large effect on LLPS in many systems *in vitro* and *in vivo* (Harmon et al., 2017a). Coacervation of globular proteins, such as lysozyme and crystallins, has been reported, but is less common than coacervation of IDPs and other chain-like biomolecules (Ishimoto and Tanaka, 1977; Thomson et al., 1987; Thurston, 2006). Patchy colloid models have been used to describe the phase behavior of various globular proteins successfully (Vlachy et al., 1993; Lomakin et al., 1999; Thurston, 2006; Liu et al., 2007; Kastelic et al., 2016; Nguemaha and Zhou, 2018; Zhou et al., 2018). In general, the condensed liquid phase formed by globular proteins is much denser than coacervates formed by IDPs, and has distinctly different physicochemical characteristics. As a result, the assembly and partitioning behavior of globular protein coacervates, and their impact on biochemical reaction are different from IDP-based coacervates. This review focuses primarily on the assembly of MLOs of chain-like biomolecules.

A feature of the phase diagram (Figure 1A) that plays a prominent role in cells to control MLO formation is the saturation concentration (ϕ_*d*_) at which condensates start to form. Many IDPs are believed to exist close to their respective saturation concentration in the cell, and subtle changes in concentration, mixing ratio or mutual interaction through biochemical modifications or binding to regulatory proteins can shift the binodal, and tip the balance to condensation (Figures 1A–C). Post-translational modifications (PTMs) that affect the charge or charge distribution of amino acid residues are an obvious mechanism to control a biomolecule's condensation propensity (Figure 1D). Indeed, serine, threonine, and tyrosine phosphorylation (Reineke et al., 2017; Rai et al., 2018), arginine methylation (Nott et al., 2015), and lysine acetylation (Cohen et al., 2015; Saito et al., 2019) have all been found to affect MLO formation *in vivo*.

*In vitro* studies using coacervates allow for a more quantitative investigation of these modifications. Phosphorylation of nephrin decreases the saturation concentration for condensation with signaling proteins N-WASP and NCK from the micromolar to nanomolar regime (Li et al., 2012). In the case of Ddx4, arginine methylation increases the saturation concentration at a given temperature by two orders of magnitude, resulting in dissolution of droplets (Nott et al., 2015). Citrullination converts arginine into neutral citrulline, which also inhibits aggregation of various disordered proteins, such as FUS, EWS, and TAF15 (Tanikawa et al., 2018). Based on these insights, the first synthetic cytomimetic organelles have recently been developed, in which kinase-mediated phosphorylation not only controls the formation of droplets but also the rate of growth (Aumiller and Keating, 2016; Nakashima et al., 2018).

More advanced control over MLO formation and localization can be achieved through protein-RNA interactions (Figures 1E,F). Nucleolus assembly, for example, starts with condensation of numerous small droplets, which coalesce to form nucleoli. However, rapid coarsening is not observed when rRNA transcription is inhibited (Berry et al., 2015; Falahati and Wieschaus, 2017). By using FIB-1 model coacervates, rRNA, which is one of the key nucleolar components, was found to expand the two-phase region toward higher salt concentration by stabilizing FIB-1 interactions, which can be interpreted as an increase of the effective interaction parameter χ (Figure 1E) (Berry et al., 2015). This mechanism of RNA-induced organelle formation has recently been exploited *in vitro* to compartmentalize mRNA directly after transcription in a cytomimetic environment by condensation with cationic peptides, or to dissolve existing peptide/RNA coacervates by overcharging them with mRNA (Figures 1C,E) (Banerjee et al., 2017).

In the case of P-granules, condensation of the granule component MEG-3 with RNA is suppressed locally in a gradient of MEX-5, which binds competitively to RNA, and lowers the RNA concentration to below the saturation level for granule assembly (Smith et al., 2016). Formation of stable P-granules *in vivo* not only requires RNA but also the helicase LAF-1, which was found to phase separate *in vitro* without RNA (Elbaum-Garfinkle et al., 2015). Paradoxically, RNA dilutes coacervates of pure LAF-1, and reduces the viscosity without changing the saturation concentration (Figure 1F). This is explained by an increased three-body repulsion in the coacervate, an effect that is not included in the simplified Flory-Huggins model above (Wei et al., 2017).

Finally, the multitude of components in P-granules and other MLOs has been found to lead to the formation of multiple phases that display mutual affinity but do not mix, both *in vivo* and *in vitro* (Feric et al., 2016; Smith et al., 2016). How the order of condensation is controlled, how different phases influence the phase behavior of others, and what the effect of macromolecular crowding is on condensation, is still poorly understood. New, multicomponent coacervate models are essential to address these questions, and to corroborate modern theories for MLO formation beyond the single-component mean-field models used thus far.

## Partitioning and Sequestration of Client Molecules

Besides the phase-separating biomolecules that “define” MLOs (hosts), there is a wide range of additional molecules (clients) that are spontaneously taken up into preformed MLOs by partitioning or sequestration, like in P-granules. Although such client molecules are not bound to the MLOs by a membrane, and can freely move in and out, it is likely that partitioning affects their availability to biochemical reactions outside the MLOs. At the same time, the local polarity, crowding and presence of other (host) biomolecules can affect client reactivity inside MLOs as well (Elbaum-Garfinkle et al., 2015; Mitrea and Kriwacki, 2016) Understanding the principles that govern partitioning is therefore essential to explain the function of MLOs (Ditlev et al., 2018). The distinction between hosts and clients is not always sharp, and clients that reach high concentrations inside MLOs have been found to significantly affect the phase diagram of the original hosts (Ditlev et al., 2018; Nguemaha and Zhou, 2018). Here, we focus on the case where client concentrations remain sufficiently low, and investigate client distribution from a partitioning point of view.

**Figure 2:**
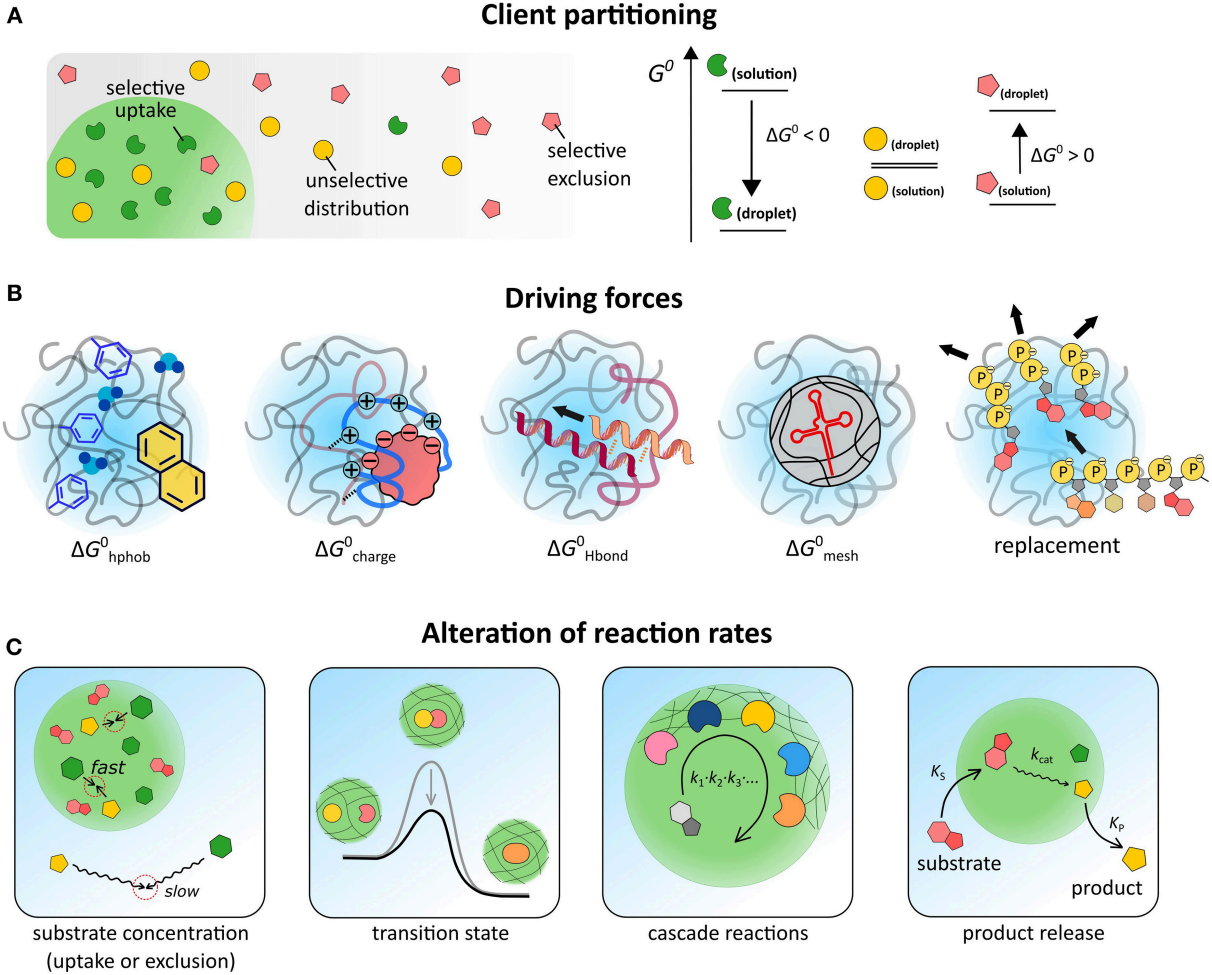
**(A)** Schematic illustration of three scenarios for partitioning, depending on the relative free energy levels of the client molecule in both phases. **(B)** Illustration of five contributions to partitioning. **(C)** Possible effects of coacervate-based compartments on reaction kinetics.

The distribution of a solute between two coexisting liquids, like the cytosol and the membraneless organelles in the cell, or the dilute phase and the coacervate droplets in a cytomimetic model, is governed by the relative standard free energy of the solute in the different phases (Figure 2A).

(2) $$\begin{array}{r} {\text{A}}_{\left(\text{α}\right)}⇄\;A_{\left(\beta \right)},\;\frac{{\text{c}}_{\text{α}}}{{\text{c}}_{\text{β}}}=K_{\text{part}}≅\lambda e^{-\Delta G^{0}/RT} \end{array}$$

where *c*_α_ and *c*_β_ are the concentration of a solute A in the coacervate and the dilute phase, respectively, *K*_part_ is the partitioning coefficient and λ is a correction factor that accounts for differences in activity between both phases. The standard molar Gibbs free energy difference of the solute between the two phases (Δ*G*^0^) sets the degree of partitioning, and is generally composed of multiple contributions: $\Delta G^{0}=\Delta G_{\text{hphob}}^{0}+\Delta G_{\text{charge}}^{0}+\Delta G_{\text{Hbond}}^{0}+\Delta G_{\text{mesh}}^{0}+...$, which we will discuss separately (Figure 2B).

The first term accounts for the fact that the local polarity inside coacervates and MLOs is usually lower than the surrounding aqueous solution. Models for the salt tolerance of coacervates provide estimates of the relative permittivity of coacervates between 45 and 60, which is explained by the presence of hydrophobic elements (e.g., amino acids residues, polymer backbone) and strongly bound hydration water (Spruijt et al., 2012; Williams et al., 2012; Nott et al., 2015). The solvation free energy in this environment ($\Delta_{\text{Ghphob}}^{0}$) is the principal driving force for partitioning of most hydrophobic solutes, such as Nile red and bromothymol blue (*K*_part_ ≈ 10^2^) (Zhao et al., 2017). Interestingly, unfolded proteins, which have their hydrophobic cores exposed, showed partitioning in PDDA-PAA coacervates with *K*_part_ > 1, as expected, but to a lower extent than native proteins, suggesting that additional contributions also play a role (Martin et al., 2016).

Many IDPs and coacervate-forming polymers contain extensive charged regions. The interaction with these charged regions ($\Delta G_{\text{charge}}^{0}$) is likely to be the main driving force for partitioning of the majority of biomolecules. The entropically favored release of bound counterions upon complexation accounts for a significant part of $\Delta G_{\text{charge}}^{0}$. In Ddx4 droplets, both positively and negatively charged proteins are selectively taken up, while neutral proteins are excluded. Small, highly charged proteins, such as lysozyme, are also readily incorporated into PDMAEMA-PAA coacervates, reaching concentrations up to 150–200 g/L (Lindhoud and Claessens, 2016), which is close to the total cytosolic macromolecule concentration. Partitioning does not seem to affect the secondary structure of globular proteins (Black et al., 2014), or enzymatic activity (Lindhoud et al., 2010; Martin et al., 2016; Kojima and Takayama, 2018), although the precise effect of coacervates on reaction kinetics is still not fully understood, as we discuss below.

Besides charge complexation, solutes can also interact with the coacervate matrix through hydrogen bonding ($\Delta G_{\text{Hbond}}^{0}$). Nucleic acids in particular may form base pairs with complementary sequences in model MLOs. Poly-U-spermine coacervates, a simple model for nucleotide-protein droplets, are able to selectively concentrate oligonucleotides and oligopeptides. For such coacervates, poly-A has a partitioning coefficient two orders of magnitude higher than poly-N or poly-U, because of base-pairing interactions (Frankel et al., 2016). However, a similar system, based on poly-U and the peptide RRASLRRASL, does not distinguish between poly-A and poly-N: both are highly concentrated inside coacervates, most likely because charge complexation dominates this partitioning (Aumiller and Keating, 2016).

To accommodate large and rigid biomolecules, including base-paired nucleic acid duplexes, the mesh of IDP or polymer chains must be deformed significantly, which disfavors partitioning ($\Delta G_{\text{mesh}}^{0}$) and destabilizes coacervates (André and Spruijt, 2018). This effect of mesh deformation can result in selectivity for small and flexible nucleic acids, and even in forced melting of DNA duplexes. Ddx4 droplets were found to concentrate single-stranded RNA and DNA ($\Delta G_{\text{hphob}}^{0}$ + $\Delta G_{\text{charge}}^{0}$), while excluding double-stranded DNA of the same length and inducing strand dissociation of shorter DNA duplexes (Nott et al., 2016). Whether a similar mechanism underlies selectivity of certain RNA bodies in cells remains to be seen (Langdon et al., 2018).

In some cases, client molecules are taken up by replacing other species in the coacervates. Although this displacement no longer qualifies as simple partitioning, it can have a very similar strong concentrating effect. In PAH-ATP droplets, RNA is concentrated by a factor 10^5^ (Frankel et al., 2016). As a single RNA chain can replace multiple nucleotides, this exchange is driven by a significant increase in entropy. The same mechanism accounts for the uptake of many polymers and colloids in polylysine-ATP droplets (Koga et al., 2011).

In cells, partitioning of biomolecules in MLOs is often more selective than *in vitro*. Specific interactions between binding domains in IDPs and client molecules, such as tubulin, may partly explain this (Jiang et al., 2015). In addition, all interactions discussed above cumulate in MLOs, and their balance is different for every client. Finally, it is important to also look beyond concentrations, and take into account the actual number of molecules available inside or outside MLOs: for low-copy-number biomolecules, stochastic effects come into play (Hansen et al., 2016), and even weak partitioning can drastically alter the cellular fate.

## Biochemical Reactions Inside Liquid Compartments

With the dynamic assembly and selective partitioning in mind, three prospects emerge for MLOs modulating biochemical reactions: (1) they may catalyze reactions that are inefficient in the cytosol; (2) they may sequester and protect key molecules from undesired reactions; or (3) they have no function *per se*, but are instead merely a consequence of the cytosolic composition (Banani et al., 2017). Experimental evidence for both enhanced reactivity (1), and reaction quenching (2) has been found in specific cases (Aguilera-Gomez and Rabouille, 2017; Alberti, 2017), but a general picture of how chemical reactivity is different inside MLOs and coacervate models is still lacking.

For a bimolecular reaction (Equation 3), reaction rates inside MLOs could differ from those in bulk solutions for two main reasons: the local concentration of reactants A and B inside MLOs may be different from outside, and the rate constant *k* may be affected by their unique environment (Equation 3, Figure 2C):

(3) $$\begin{array}{l} A_{\;(\alpha )}+B_{\;(\alpha )}\to C_{\;(\alpha )}⇄C_{\;(\beta )},\\ \frac{d[C]}{dt}=k[A][B]=k_{0}(t,T)\;e^{-\frac{\Delta G^{‡}}{RT}}[A](t)\;[B](t) \end{array}$$

The concentration effect is straightforward and usually contributes to higher reaction rates, as a wide range of solutes is found to accumulate inside coacervates (see previous section). The effect of *k* is less obvious and much more interesting, as reactions may be either diffusion-limited (*k*_0_) or transition-state-limited (Δ*G*^‡^) (Figure 2C). Moreover, in heterogeneous and crowded environments, such as coacervates, *k* generally becomes a time-dependent quantity and the distribution of reactants and the tortuosity of the reaction path must be taken into account (Minton, 2006; Bénichou et al., 2010; Tabaka et al., 2014).

A general limitation in elucidating fundamental principles of reactivity inside coacervates is that concentrations of the individual components are often not quantified, and kinetics is not measured in both phases separately. Experiments suggest that many enzymatic reactions involving small molecule substrates are accelerated inside coacervates, primarily because of enhanced substrate and cofactor concentration (Koga et al., 2011; Kojima and Takayama, 2018; Stroberg and Schnell, 2018). Hexokinase partitions inside polylysine-ATP droplets with *K*_part_ ≈ 20, and its activity is enhanced 2-fold, because of high local ATP and Mg^2+^ concentrations (Koga et al., 2011). Lipase activity is increased about 2-fold in coacervate micelles, because of a combination of substrate concentration and stabilization of the enzyme's active form (Lindhoud et al., 2010).

Hammerhead ribozyme activity has also been studied in liquid compartments. In dextran droplets, substrate cleavage is about 70 times faster than in solution, which was attributed to an increased ribozyme (*K*_part_ ≈ 3,000) and substrate (*K*_part_ ≈ 40) concentration (Strulson et al., 2012). A 60-fold decrease of reaction rate was measured for the same ribozyme in polylysine-CMDex coacervates, despite an enhanced concentration, suggesting that the physicochemical details of the coacervate environment also impact reactivity (Drobot et al., 2018). The biphasic kinetics in the latter coacervates indicate that catalysts, such as ribozymes or enzymes, may exist as distinct populations in MLOs.

A more detailed analysis of the effect of the coacervate environment on reactivity is complicated, because both diffusion and the energy landscape can be affected by confinement in MLOs, and in either direction. Macromolecular crowding and strong interactions inside the droplets (Figure 2C) can lead to anomalous, often reduced diffusion (Menjoge et al., 2008; Kausik et al., 2009; Shakya and King, 2018), thus contributing to slower kinetics. However, those same effects can also favor a more active enzyme conformation or lower the energy barrier, resulting in a higher rate constant, or they could trap an enzyme in an inactive form, resulting in a vanishing reactivity. A point in case is the cell-free gene expression and folding of fluorescent reporter proteins: inside PEG-based coacervates, transcription was found to take place with a two orders of magnitude higher polymerase association constant and a 6-fold higher transcription rate constant (Sokolova et al., 2013). However, in polylysine-CMDex coacervates, gene expression appeared to be slower overall, and the yield was reduced significantly by protein aggregation in the coacervates (Dora Tang et al., 2015). Apparently, chemical interactions sometimes have a larger effect than macromolecular crowding, although a detailed analysis requires more systematic studies using benchmark reactions.

For more complex processes, including multi-step reactions and reaction networks, coacervates could further affect the kinetics. The coacervate matrix can act as scaffold to spatially organize enzymatic cascades, and enhance overall processivity (Figure 2C) (Klingauf et al., 2006; Davis et al., 2015; Kastritis and Gavin, 2018). Such a functional role has been proposed for example for nucleoli and processing bodies. Finally, differential partitioning of substrates and products of a reaction could result in an effective rate acceleration (Figure 2C), akin to what happens in phase transfer catalysis. The uptake of a fusion protein with one or more LAF-1-derived RGG domains and subsequent release of a cargo domain after cleavage from the fusion protein inside coacervates provides a promising example, although rates have not been determined in this case (Schuster et al., 2018).

## Outlook

This review has focused on cytomimetic approaches to address three aspects of MLOs that are still poorly understood: dynamic assembly, partitioning of client molecules and reaction kinetics inside MLOs. Coacervates serve as model systems to investigate these aspects systematically *in vitro*. However, most coacervates are still far from resembling cellular MLOs, and significant progress is needed to develop coacervate-based cytomimetic systems that capture the full complexity of spatiotemporal organization in cells. Multicompartment coacervates have recently been developed based on ELPs with different chain lengths (Simon et al., 2017), and different IDPs derived from nucleoli (Feric et al., 2016), in an attempt to better understand the hierarchical organization of the numerous different components found in many MLOs. A related aspect that has not been experimentally addressed yet, is how different types of coacervates or MLOs could coexist in the same cytosol, without mixing (Feric et al., 2016; Harmon et al., 2017b), as has long been known for many other multicomponent liquid mixtures (Mace et al., 2012; Torre et al., 2014). This could be connected to amphipathic biomolecules that adsorb at the liquid-liquid interface to stabilize it (Mason et al., 2017; Simon et al., 2017), or to a continuous turnover of coacervate material, away from thermodynamic equilibrium, in order to suppress Ostwald ripening (Zwicker et al., 2015). Such aspects represent the oncoming challenges on the road to artificial organelles and cells.

## Author Contributions

All authors listed have made a substantial, direct and intellectual contribution to the work, and approved it for publication.

### Conflict of Interest Statement

The authors declare that the research was conducted in the absence of any commercial or financial relationships that could be construed as a potential conflict of interest.
